# Nonstructural p26 proteins encoded by the 3’-proximal genes of velariviruses and criniviruses are orthologs

**DOI:** 10.1007/s00705-019-04491-8

**Published:** 2019-12-11

**Authors:** I. B. Rogozin, A. A. Agranovsky

**Affiliations:** 1 National Center for Biotechnology Information, National Library of Medicine, National Institutes of Health, 8600 Rockville Pike, Bethesda, MD 20894 USA; 2 Faculty of Biology, Moscow State University, Moscow, 119991 Russia; 3 Center of Bioengineering, Russian Academy of Sciences, Moscow, Russia

## Abstract

The 3’-most genes in RNA-2 of the *Crinivirus* genus members (family *Closteroviridae*) code for non-structural p26 proteins that share amino acid sequence similarity [Stewart LR, Hwang MS, Falk BW (2009) Virus Res 145:293-299]. In this study, sensitive bioinformatic tools have been used to identify the homologous p26 proteins encoded by the 3’ genes in monopartite genomes of the members of *Velarivirus*, another *Closteroviridae* genus, and mint vein banding-associated virus, an unassigned member of the family. The p26 proteins showed similarity in their predicted secondary structures, but an amino acid sequence alignment showed no strictly conserved positions, thus indicating a high plasticity of these non-structural proteins. The implications of the sequence analysis for possible functions of the crinivirus and velarivirus p26 proteins are discussed.

**Electronic supplementary material:**

The online version of this article (10.1007/s00705-019-04491-8) contains supplementary material, which is available to authorized users.

The family *Closteroviridae* includes about 50 filamentous plant viruses with large positive-sense RNA genomes that show numerous traces of recombination events, such as gene duplication and gene capture [1–3]. Closteroviruses contain up to 12 genes, most of which are arranged in two conserved modules: the replicative module, which encodes proteins responsible for RNA synthesis and membrane modification, and the five-gene block, which encodes proteins involved in particle formation and cell-to-cell movement [2–4] (Fig. 1). In addition, closteroviruses carry variable accessory genes in the 3’ part of their genome (Fig. 1). Some products of the 3’ genes are conserved in some members of the *Closteroviridae* [3, 5], whereas others have no apparent homologs and are species-specific.

**Fig. 1:**
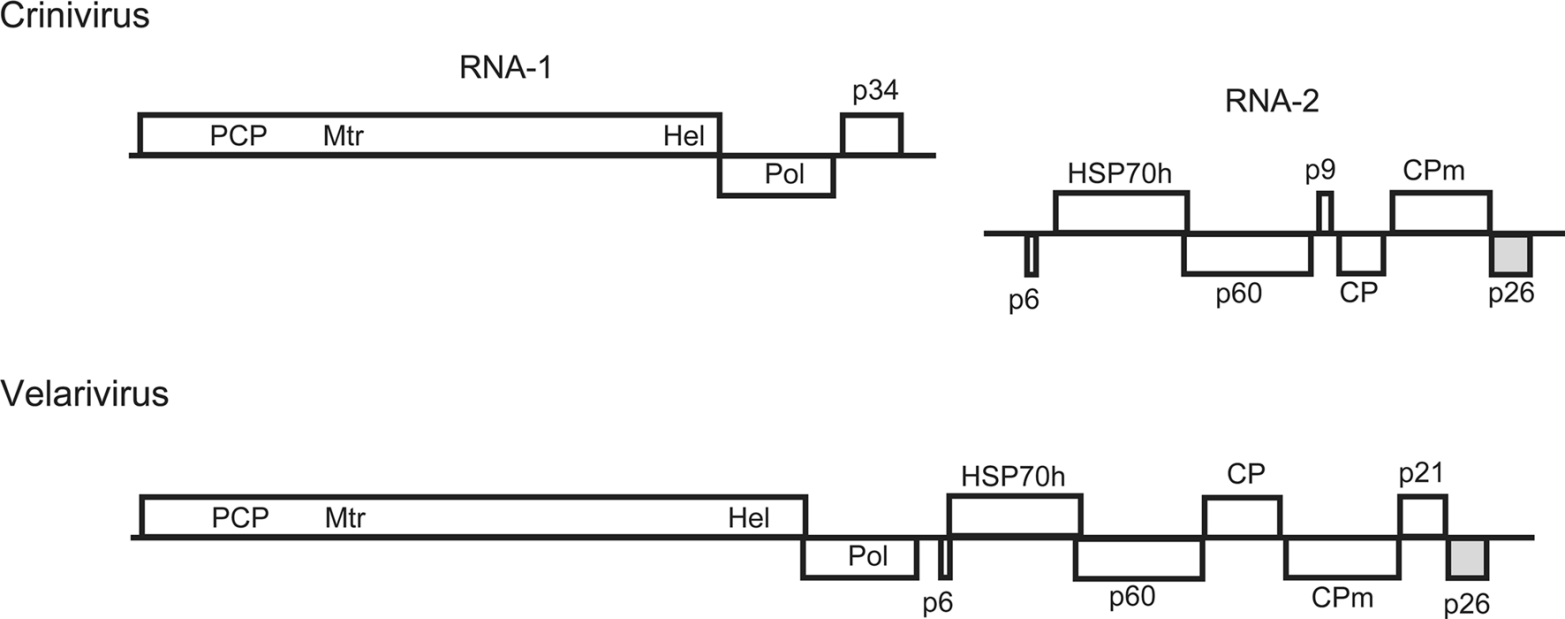
Genome maps of representatives of the genera *Crinivirus* (lettuce infectious yellows virus, LIYV) and *Velarivirus* (little cherry virus 1, LChV-1) drawn approximately to scale. The arrow indicates the RNA 3’ end. ORFs are shown as boxes. Encoded domains: PCP, papain-like cysteine proteinase; Mtr, methyltransferase; Hel, helicase; Pol, RNA polymerase. Proteins: p6, small hydrophobic protein; HSP70h, HSP70-related protein; p60, ~ 60 kDa proteins; CP and CPm, major and minor coat proteins, respectively. The ORFs for p26 proteins are shaded

The members of the genus *Crinivirus* have bipartite genomes with RNA-1 and RNA-2 bearing the replicase module and the five-gene block, respectively, plus some additional 3’ ORFs [6, 7] (Fig. 1). The 3’ genes in RNA-2 of criniviruses code for proteins with a molecular weight of about 26 kDa (p26) that have certain similarity in amino acid sequence and predicted secondary structure [3, 5] (Fig. 1). In this study, our purpose was to identify the p26-related proteins in members of the other genera and unassigned virus species of the family *Closteroviridae*.

The p26 protein sequences (Supplementary Table S1) were downloaded from the Refseq database using BLASTp and PSI-BLAST searches (www.ncbi.nlm.nih.gov) [8]. Multiple alignments were produced with the T-Coffee program [9]. HMMER2.0 toolbox [10] was used for Hidden Markov Model (HMM) reconstruction and sequence comparisons, HHpred [11] for HMM profile comparisons, and JPRED4 [12] for secondary structure predictions.

Initial BLAST and PSI-BLAST searches did not reveal any putative p26 protein homologs outside the genus *Crinivirus* when the crinivirus p26 sequences were used as a query (Supplementary Table S1). At the next step, we used HMMER 2.0, a sensitive tool for detecting remote protein homologs [10]. The hidden Markov model for the crinivirus p26 proteins was constructed and used for directed search for the 3’ ORF products in members of other *Closteroviridae* genera (*Velarivirus*, *Closterovirus*, and *Ampelovirus*). Low probability values (indicating a statistically significant similarity) were obtained for the 27- to 29-kDa proteins encoded by the 3’-most ORFs of velariviruses (below, also referred to as p26 proteins) (Table 1). Figure 2 shows a sequence alignment of the p26 proteins of criniviruses and velariviruses. Although some positions in the alignment are occupied by similar amino acid residues, none of them is strictly conserved (Fig. 2). Analysis of secondary structure suggests that the p26 proteins of velariviruses and criniviruses are alpha-helical with a few beta-strands (Fig. 2), which corroborates the previous data for the crinivirus proteins [5]. Six alpha-helices in the p26 proteins have a similar location (Fig. 2), suggesting that these proteins share a common three-dimensional structure.

**Table 1:** Significance of similarities between the crinivirus p26 hidden Markov model and the velarivirus p26 proteins

Sequence ID	Score	E-value
Cordyline virus 1 ADU03662	-141.2	0.066
Cordyline virus 2 AFJ05053	-138.7	0.049
Cordyline virus 3 AGF73886	-126.8	0.012
Cordyline virus 4 AGF73893	-136.9	0.04
Grapevine leafroll-associated virus 7 AEQ59451	-130.0	0.018
Little cherry virus-1 CEO12417	-125.1	0.0098

**Fig. 2:**
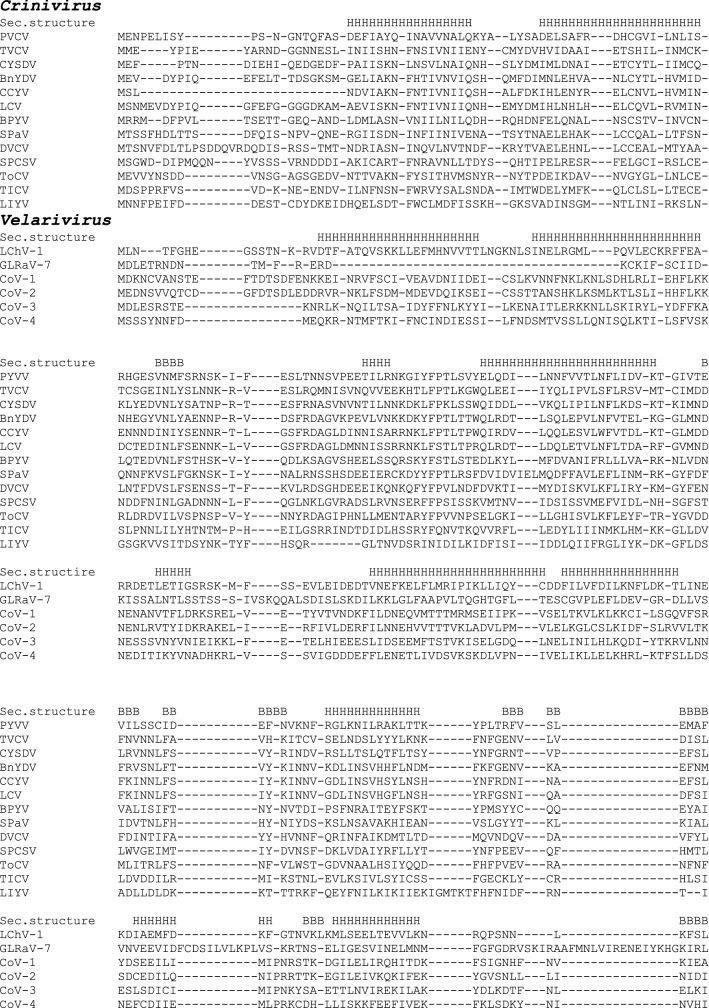

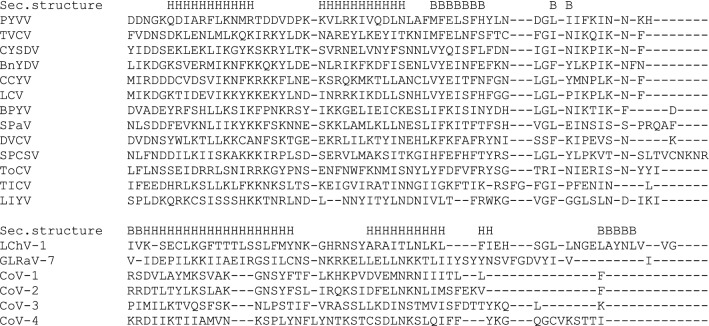
Multiple alignment and predicted secondary structure of the p26 proteins of representatives of the genera *Crinivirus* and *Velarivirus*. Predicted alpha-helices (H) and beta-strands (B) are indicated. Criniviruses: PYVV (potato yellow vein virus, YP_054414.1), TVCV (tetterwort vein chlorosis virus, ALE18225.1), CYSDV (cucurbit yellow stunting disorder virus, NP_851578.1), bean yellow disorder virus (BnYDV, ABY66971.1), CCYV (cucurbit chlorotic yellows virus, YP_006522433.1), LCV (lettuce chlorosis virus, YP_003002364.1), BPYV (beet pseudo-yellows virus, AAQ97392.1), SPaV (strawberry pallidosis-associated virus, YP_025091.1), DVCV (diodia vein chlorosis virus, ADU25040.1), SPCSV (sweet potato chlorotic stunt virus, AEO37527.1), ToCV (tomato chlorosis virus, AJY78063.1), TICV (tomato infectious chlorosis virus, YP_003204962.1), LIYV (lettuce infectious yellows virus, NP_619699.1). Velariviruses: LChV-1 (little cherry virus 1, acc. CEO12417.1), GLRaV-7 (grapevine leafroll-associated virus 7, acc. AEQ59451.1), CoV-1 (cordyline virus 1, acc. ADU03662.1), CoV-2 (cordyline virus 2, AFJ05053.1), CoV-3 (cordyline virus 3, AGF73886.1), CoV-4 (cordyline virus 4, AGF73893.1)

We also performed an additional HMM database search using the combined multiple alignment of crinivirus and velarivirus p26 proteins (Fig. 2) as a query for HMMER2.0. The 24-kDa protein of mint vein banding-associated virus (MVBaV), an unassigned member of the family *Closteroviridae* [13], was detected as a possible remote homolog (Fig. 3). A significant probability value (0.009) was obtained, supporting the relatedness of the MVBaV p24 to the p26 of criniviruses and velariviruses (Fig. 3). Attempts to include the MVBaV p24 into the multiple alignment using T-Coffee [9] were not successful due to the lack of detectable similarity in the C-terminal regions (Fig. 3). Additional HHPred database searches did not reveal any putative homologs of crinivirus/velarivirus p26 proteins among the available HMM profiles [11].

**Fig. 3:**
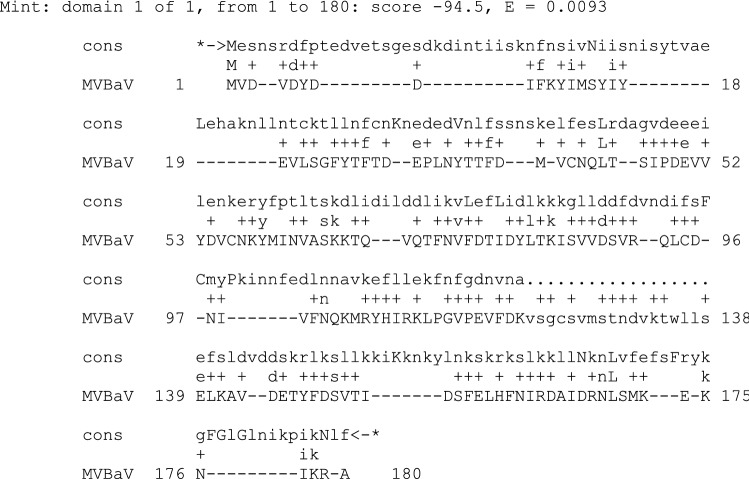
Sequence alignment between the hidden Markov model consensus sequence for the crinivirus and velarivirus p26 proteins and a remote homolog, mint vein banding-associated virus 24-kDa protein (NC_038420.1). Due to the lack of detectable similarity in the C-terminal regions, only partial sequences are shown

The data presented here indicate that the p26 genes are not *Crinivirus* taxon-specific as has been thought but are conserved across the monopartite genomes of *Velarivirus* members and mint vein banding-associated virus. Taking into account the 3’-proximal location of the p26 genes (Fig. 1), similarity of the predicted secondary structures, and statistically significant similarity of the amino acid sequences (Table 1, Fig. 2), it is likely that the p26 proteins of criniviruses and velariviruses are orthologs that may perform the same or similar function(s). It should be noted that members of the genera *Crinivirus* and *Velarivirus* are markedly different from each other in their biological properties and the genome structure. Criniviruses have divided genomes, are transmitted by whiteflies, and infect herbaceous hosts, whereas velariviruses possess monopartite genomes, have no known vectors, and infect woody hosts [2] (Fig. 1). The absence of conserved amino acid positions in the p26 alignment suggests high plasticity of these non-structural proteins, which may indicate their involvement in the response of virus systems to rapidly changing environmental conditions. On the other hand, the p26 protein of lettuce infectious yellows virus (LIYV), the type member of the genus *Crinivirus*, induces specific ultrastructures in the infected cells – conical plasmalemma deposits over plasmadesmata – that are thought to be associated with the vascular transport of the virus [7, 14, 15]. In support of this, a knockout LIYV p26 mutant proved to be unable to spread systemically in a *Nicotiana benthamiana* host [15]. Although the *Closteroviridae* members other than LIYV do not induce plasmalemma deposits, the involvement of crinivirus and velarivirus p26 proteins in systemic transport cannot be excluded and needs to be tested experimentally.

## Electronic supplementary material

Below is the link to the electronic supplementary material.

Supplementary material 1 (DOC 19 kb)
