# Supplementary material for: Nonstructural p26 proteins encoded by the 3’-proximal genes of velariviruses and criniviruses are orthologs
Source: Arch Virol. 2019 Dec 11;165(2):439–43. doi: 10.1007/s00705-019-04491-8 (PMC6994434; doi:10.1007/s00705-019-04491-8)
Supplement: Supplementary file 1 — Supplementary material 1 (DOC 19 kb) [file 705_2019_4491_MOESM1_ESM.doc]

Supplementary Table S1. Sequences used in this study.

Criniviruses

1 PotatoYellowCRINI|50428957|ref|YP_054414.1|/1-230 hypothetical protein PYVVs3_gp3 
2 TetterwortVein|ALE18225.1/1-229 26.9-kDa protein 
3 CucurbitYellowStunt|30840976|ref|NP_851578.1|/1-228 p26 
4 BeanYellow|164699046|gb|ABY66971.1|/1-231 p27 
5 CucurbitChlorotic|395865417P_006522433.1|/1-213 hypothetical 26-kDa protein 
6 LettuceChlorosis|242916050|ref|YP_003002364.1|/1-233 
7 BeetPseudoyellows|37677507|gb|AAQ97392.1|/1-230 p26

8 StrawberryPallidosis|48696549|ref|YP_025091.1|/1-239 p28 
9 DiodiaVein|315451923|gb|ADU25040.1|/1-238 p28 
10 SweetPotatoChlorotic|346642218|gb|AEO37527.1|/1-242 p28 
11 TomatoChlorosis|AJY78063.1/1-232 p27 
12 TomatoInfectiousChlorosis|258676972|ref|YP_003204962.1|/1-228 p27 
13 Lettuce infectious yellows p26   NP_619699

Velariviruses  
14 GrapevineLeafroll7_VELARI|351735571|gb|AEQ59451.1|/1-225 P25 
15 LittleCherry1|CEO12417.1/1-231 
16 Cordyline1|315274223|gb|ADU03662.1|/1-221 p26 
17 Cordyline2|386305448|gb|AFJ05053.1|/1-223 25.9 kDa 
18 Cordyline3|451898835|gb|AGF73886.1|/1-217 25.4 kDa protein 
19 Cordyline4|451898846|gb|AGF73893.1|/1-222 25.8 kDa protein

Additional distant homologs

20 areca palm velarivirus 1,YP_009140439.1,p26

21 Mint vein banding-associated virus NC_038420.1,3’-gene product, 24K
